# E-Cadherin Regulates HIF1-α In Vitro in Induced 3D Spheroid Models of Human Breast Cancer Through Both mTOR and Notch1 Signaling

**DOI:** 10.3390/biomedicines13122890

**Published:** 2025-11-26

**Authors:** Yin Ye, Dollada Srisai, Sanford H. Barsky

**Affiliations:** Department of Pathology, Anatomy and Cell Biology, and the Clinical and Translational Research Center of Excellence, Meharry Medical College, 1005 Dr. D.B. Todd Jr. Boulevard, Nashville, TN 37208, USA

**Keywords:** 3D spheroidgenesis, induced spheroidgenesis, spontaneous spheroidgenesis, HIF-1α, PI3K/AKT/mTOR signaling, Notch1 signaling, calpain, E-cadherin proteolysis

## Abstract

**Background**: Both spontaneous and induced 3D spheroid models are among many in vitro models that recapitulate aspects of in vivo cancers. Although numerous studies have described the spatiotemporal relevance of these 3D models, there has been a paucity of studies investigating the signaling pathways that are activated during spheroidgenesis. **Methods**: Since in vitro 3D spheroidgenesis is thought to reflect at least some of the in vivo aspects of cancer biology (which undoubtedly involve cell adhesion, metabolism, and hypoxia-related pathways) and since we previously investigated these pathways in a model of spontaneous spheroidgenesis, this present study investigates these pathways in a model of induced spheroidgenesis with comparative studies involving a series of well-known E-cadherin-positive (MCF-7, HTB-126, HTB-27) and E-cadherin-negative (MDA-MB-468, MDA-MB-231, BT-549) human breast carcinoma cell lines. **Results**: Our findings demonstrate that during early induced spheroidgenesis, E-cadherin regulates hypoxia-inducible factor 1-alpha (HIF-1α) predominantly through PI3K/AKT/mTOR signaling and to a lesser extent through Notch1 signaling. Both the knockout of E-cadherin and calpain-mediated E-cadherin proteolysis result in a remarkable reduction in HIF-1α. **Conclusions**: 3D spheroid models recapitulate, in part, some of the in vivo stages of cancer progression, which include primary tumor clusters, lymphovascular emboli, and micrometastases, the signaling pathways present in these 3D spheroid models likely have relevance in vivo.

## 1. Introduction

Three-dimensional spheroid models are one of many in vitro models that recapitulate both structural and functional aspects of in vivo cancers. Many of these in vitro models have gained in popularity over the past decade as they provide many inherent advantages for drug screening and other translational research approaches [1]. Three-dimensional spheroid models recapitulate key features of solid tumors, bridging the gap between traditional 2D cell cultures and complex in vivo models [2,3,4,5,6,7,8,9]. Numerous studies have described the spatiotemporal relevance of these 3D models [10,11,12,13,14,15,16,17,18,19].

Our initial studies focused on a 3D model of spontaneous spheroidgenesis, which consisted of studies of a patient-derived xenograft (PDX) model of inflammatory breast cancer (IBC) called Mary-X, where minced fragments of the extirpated xenograft spontaneously gave rise to loose aggregates, which tightened in suspension culture to give rise to high-density 3D spheroids over 12–24 h [20,21,22,23,24,25]. Spheroids that form over this time period have been shown by Principal Component Analysis to be the in vitro equivalent of lymphovascular tumor emboli [22]. There have been only a few 3D models of spontaneous spheroidgenesis. In contrast, there have been many models of induced 3D spheroidgenesis. These later models arise from single cells originally growing as monolayers, subsequently liberated by trypsin and suspended in ultra-low attachment (ULA) plates, where the suspended cells undergo spheroidgenesis. Because induced 3D spheroidgenesis begins with single cells rather than preformed loose aggregates, we felt that the use of such induced models might be more generally relevant to tumor biology in vivo. In any case, there has been a paucity of studies that have actually investigated the signaling pathways, which are activated as a result or product of 3D induced spheroidgenesis.

Since both in vitro induced 3D spheroidgenesis and its in vivo equivalents, which include primary tumor clusters, lymphovascular emboli, and micrometastases, undoubtedly involve common cell adhesion, growth, metabolism, and hypoxia-regulating pathways [26,27,28,29,30], and because our previous studies in spontaneous spheroidgenesis observed the roles of E-cadherin, E-cadherin proteolysis, mTOR, and Notch signaling [23,24,25], we decided to focus on these signaling pathways in our present study.

## 2. Materials and Methods

### 2.1. Institutional Approvals

Mary-X had been derived from a patient with biopsy-proven IBC in the 1990s and made into a patient-derived transplantable xenograft (PDX). Studies were originally conducted under UCLA’s Human Subject Protection and the Chancellor’s Animal Research Committee (Certification 95-127-11). The xenograft has been phenotypically stable for over 30 years of passage. Most recent animal studies were conducted at Meharry Medical College, OLAW D16-00261 (A3420-01), IACUC protocol 24-02-1443.

### 2.2. ATCC Patent Deposits and Cell Identification

Mary-X and its in vitro-derived spheroids were deposited in the ATCC cell repository (Manassas, VA, USA) as PTA-2737 and PTA-27376, respectively, and recently verified and re-verified to be both novel and human in origin (STRA4993). Histological and immunocytochemical verification of its IBC nature was further confirmed by comparing it to anonymized human breast cancer cases of IBC (IRB Protocol 23-10-1410) obtained through the Meharry Medical College In Situ Tissue -omics Core established by IRB 14-07-229.

### 2.3. Other Human Breast Cancer Cell Lines

Other human breast cancer cell lines included the E-cadherin-positive (MCF-7, HTB-126, and HTB-27) and E-cadherin-negative (MDA-MB-468, MDA-MB-231, BT-549) cell lines, all previously purchased from ATCC, Manassas, VA, USA.

### 2.4. Inhibitors, Chemicals, and Antibodies

The PI3K inhibitor LY294002, calpain inhibitor calpeptin, the Notch inhibitor DAPT, and the mTOR inhibitor rapamycin were purchased from Fisher Scientific, Waltham, MA, USA. For inhibitor treatment, the inhibitor stock solutions were made in DMSO with 1000× of working concentrations.

Antibodies used included the following: Notch Activated Targets Antibody Sampler Kit, #68309, Cell Signaling Technology (CST), Danvers, MA, USA; Hypoxia Pathway Antibody, Sampler Kit, #15792, CST, which include antibodies to HIF-2α, VHL, hydroxy-HIF-1α, and PHD-2/Egln1; mTOR Substrates Antibody Sampler Kit, #9862, CST; and E-cadherin (G-10), #sc-8426, Santa Cruz Biotechnology, Santa Cruz, CA, USA; and Calpain 2 Large Subunit (M-type) (1:1000 dilution, #3195, (CST)). Antibodies to housekeeping gene products included β-tubulin, #sc9104, Santa Cruz Biotechnology, and β-actin, #937215, R&D Systems, Minneapolis, MN, USA.

### 2.5. In Vitro Studies of Both Spontaneous and Induced Spheroidgenesis

Mary-X was placed in culture and gave rise to liberated loose aggregates in suspension culture, which spontaneously tightened into spheroids over the next 24 h and remained in suspension culture. The other human breast cancer cell lines, which were trypsin-harvested from monolayer cultures grown in Dulbecco’s Modified Eagle’s Medium (DMEM) and supplemented with 10% (vol/vol) FBS and 100 U/mL penicillin/streptomycin, could be induced to form spheroids. Briefly, 5 × 10^4^ cells were seeded on 24-well ultra-low attachment (ULA) plates under normoxic conditions to induce spheroidgenesis. Both spontaneous and induced spheroidgenesis were monitored by both phase contrast microscopy and time-lapse photography using the Lux3 camera (CytoSMART Technologies, Axion BioSystems, Atlanta, GA, USA) over a 24 h period. The induced spheroids were collected and harvested for Western blot analysis. The effects of the various inhibitors on induced spheroidgenesis were investigated in subsequent studies. The working concentrations of each inhibitor used were: LY294002: 50 μM; rapamycin: 10 µM; calpeptin: 25 µM; and DAPT: 50 µM. The inhibitors were added immediately after seeding of each of the breast cancer cell lines on the ultra-low attachment (ULA) plates. All the spheroids, with and without inhibitors, were then harvested for Western blot analysis.

### 2.6. CRISPR/Cas9-Mediated Generation of Knockout Cells

A lentivirus vector lentiCRISPRv2 puro, Addgene, #98290, Watertown, MA, USA, was used to transfect MCF-7 cells. Single Guide RNAs (sgRNAs) targeting E-cadherin were designed using CHOPCHOP, a web-based search tool of sgRNAs, and ligated into a *Bsm*BI-lineated lentiCRISPRv2 puro plasmid. The targeting sequence for E-cadherin used was AAGTCACGCTGAATACAGTG. The correct insertion of the gRNA sequence was confirmed via sequencing prior to transfection. Following transfection, the MCF-7 cells were selected with 1 µg/mL puromycin for 2 days. The surviving cells were divided and placed in 96-well plates for 15 days. The single clones were selected and analyzed for the expression levels of E-cadherin using Western blot. Finally, sequencing of gRNA-targeting sites was used to verify the correct knockout (KO) of E-cadherin. Sequencing was performed by Azenta Life Sciences, Burlington, MA, USA.

Select clones showing successful and complete E-cadherin knockout, e.g., MCF-7/gCDH1/#4 and MCF-7/gCDH1/#56, were monitored by both phase contrast microscopy, time-lapse photography, and Western blot analysis for effects on induced spheroidgenesis and associated signaling pathway alterations.

### 2.7. Western Blot Analysis

Trypsinized monolayers or induced spheroids were collected at indicated time intervals, washed in ice-cold PBS, and then suspended in Laemmli Sample Buffer, #1610737, Bio-Rad, Hercules, CA, USA, supplemented with β-mercaptoethanol, and then boiled for 10 min. Whole-cell lysates were separated by sodium dodecyl sulfate–polyacrylamide gel electrophoresis on precast 4 to 20% Mini-Protean TGX gels, Bio-Rad, transferred to PVDF membranes, and probed with the indicated antibodies.

**Figure 1:**
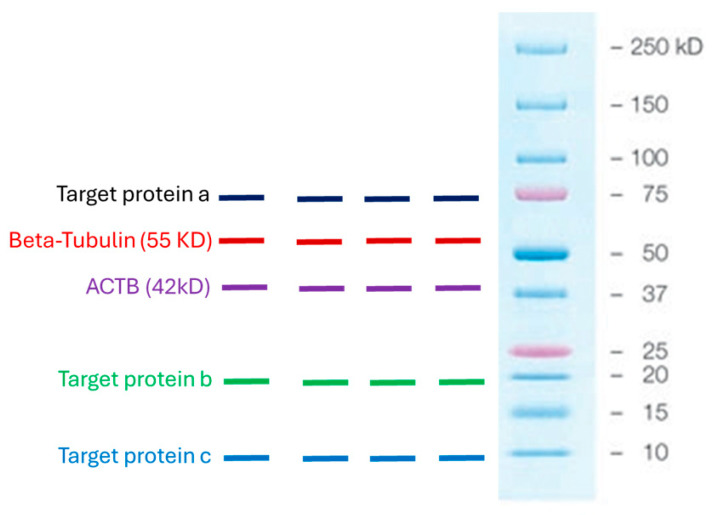
Graphical demonstration of the molecular weights of the housekeeping proteins, β-tubulin and actin-B, used in the study, and the molecular weights of some of the targeted proteins to illustrate that there should be a reasonable distance between the housekeeping protein and the targeted protein on the membrane so that when it is cut into strips, their relative band intensities can be clearly visualized and quantitated.

We used housekeeping genes, β-tubulin and actin-B. These are standard housekeeping genes used to normalize Western blots. We have used these housekeeping genes interchangeably in numerous previous studies because their levels of expression remain constant despite numerous experimental manipulations [24,25]. Our experience has taught us that when showing numerous Western blots, it is necessary to use multiple housekeeping genes to account for any impact that an experimental condition may have on the expression of a single housekeeping gene. In addition, our housekeeping genes, β-tubulin and actin-B, have different molecular weights, 55 kDa and 42 kDa, respectively. Our target proteins also differ in size, and we want to use the housekeeping protein that lies furthest from the target protein on the membrane so that when the membrane is cut into strips, we can optimally distinguish the target protein from the housekeeping protein and quantitate their relative band intensities (Figure 1).

Quantification of the intensity of the protein bands was performed by using ImageJ (NIH) [https://ij.imjoy.io/ Version 1.53f, accessed on 22 August 2025]. We have used this software in previously published manuscripts, and, therefore, we are very familiar with ImageJ [24,25]. Although emerging chemiluminescence software may, in general, improve accuracy, we had to balance our experience with ImageJ with our inexperience with the emerging chemiluminescence software.

### 2.8. Statistical Analysis

All individual in vitro experiments were repeated five times. Within each experiment, five technical replicates were also conducted. We typically provide five repeats of every experiment and five replicates for any time point. This optimally controls for experimental error and variations. Representative results depicted show means. Illustrated photographs depicting phase contrast appearances of monolayer or suspension cultures were representative of our typical results. All stated or calculated differences imply differences of statistical significance, assessed by the two-tailed Student’s *t*-test as well as a one-way ANOVA, since our independent factor was time of induced spheroidgenesis under natural conditions or inhibitor conditions.

## 3. Results

### 3.1. Spontaneous v Induced 3D Spheroidgenesis Differ Regarding Their E-Cadherin Dependency

**Figure 2:**
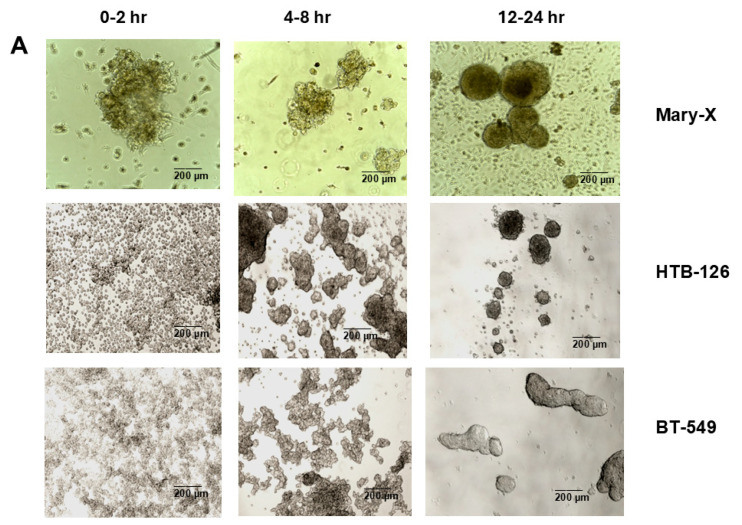

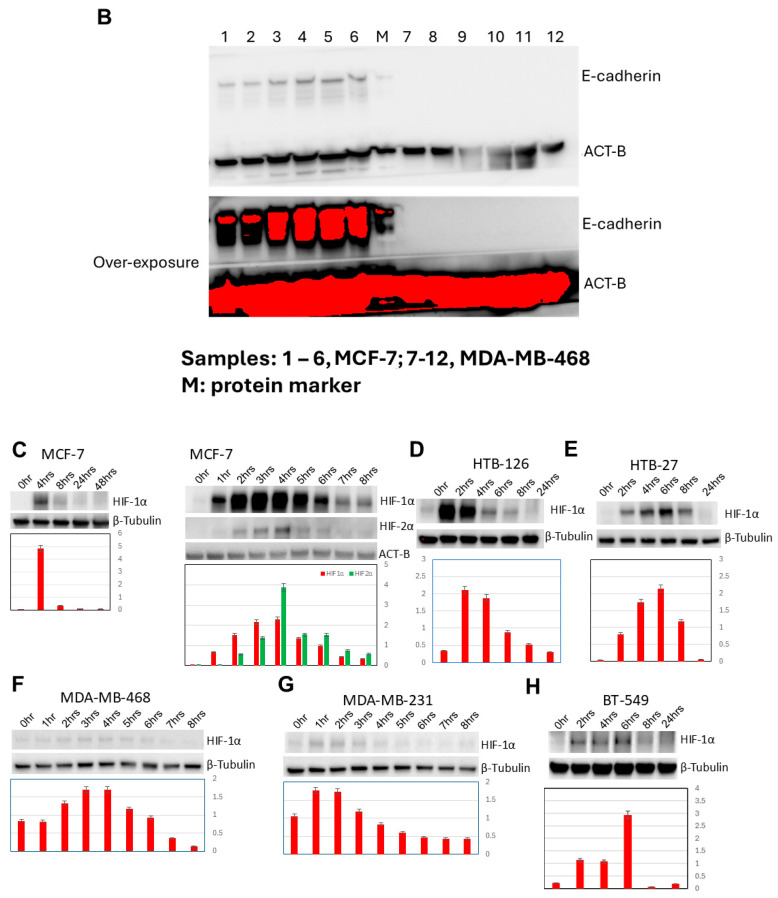
Three-dimensional spheroidgenesis, E-cadherin, and HIF-1α. The phase contrast appearance of spontaneous (Mary-X) vs. induced spheroidgenesis (HTB-126 and BT-549) along the indicated time course is similar; the end result being the production of tight high-density spheroids in suspension culture (**A**). Scale bars are provided. Western blot analysis confirms MCF-7 E-cadherin positivity but complete negativity in the MDA-MB-468 clone used in this study (**B**). Western blot analysis in induced spheroidgenesis shows an increase in HIF-1α, followed by a decrease (**C**–**H**) in both E-cadherin-positive as well as -negative cell lines, but HIF-1α levels overall are higher in the former (**C**–**E**) rather than the latter (**F**–**H**).

Mary-X represents a rare model of spontaneous 3D spheroidgenesis, which has been studied extensively in our past studies. The model, arising from minced fragments of extirpated xenografts, remains in suspension culture, does not form monolayers, and arises from loose cell aggregates as opposed to single cells (Figure 2A). The model is highly dependent on both full-length E-cadherin and one of its fragments, E-cad/NTF1, produced intracellularly by calpain-mediated proteolysis. Inhibitors of E-cadherin homodimer formation (anti-E-cadherin, trypsin, and EDTA) both inhibit and reverse spontaneous spheroidgenesis through disadherence. Spheroids that form over a 24 h time course exhibit both high density and budding.

In contrast, induced 3D spheroidgenesis arises from single cells originally growing as monolayers, which, like in spontaneous spheroidgenesis, tighten into high-density spheroids over a 24 h time course (Figure 2A). Induced 3D spheroidgenesis is not dependent on E-cadherin, as equally tight spheroids with high densities are observed in E-cadherin-negative cell lines (Figure 2A). However, we wanted to compare and contrast the signaling that characterizes early induced spheroidgenesis in E-cadherin-positive *v*-negative cell lines. We studied three well-known E-cadherin-positive (MCF-7, HTB-126, HTB-27) and three well-known E-cadherin-negative (MDA-MB-468, MDA-MB-231, BT-549) lines in all of our experiments. All of our experiments were conducted with these six cell lines. All of the E-cadherin-positive lines showed similar and equivalent findings, and all of the E-cadherin-negative lines showed similar and equivalent findings, but the E-cadherin-positive lines differed in signaling from the E-cadherin-negative lines.

### 3.2. Increased HIF-1α Expression in Induced 3D Spheroidgenesis May Be Upregulated by E-Cadherin

The E-cadherin status of all lines used in the present study was confirmed by both immunocytochemical studies and Western blot in previous studies [24,25]. Because the MDA-MB-468 had been described in the literature as being E-cadherin positive, we repeated our E-cadherin Western blot studies to verify that the clone we were using was E-cadherin negative (Figure 2B). Both E-cadherin-positive and E-cadherin-negative cell lines express HIF-1α in early induced 3D spheroidgenesis, but HIF-1α expression is significantly greater in the E-cadherin-positive lines (Figure 2C–H). Initially, when MCF-7 cells were digested by trypsin into single cells, the level of HIF-1α was low (Figure 2C). Its level then increases and peaks 4 h after seeding (*p* < 0.01), decreases after that time, and reaches a rather low level at 8 h. Its level then continues to decrease and reaches its lowest levels at 24–48 h. Both HIF-1α and HIF-1β observed over a more detailed time course reveal similar increases in both, with peaks at 4 h after seeding and then decreases (*p* < 0.01) (Figure 2C). This pattern and peak of HIF-1α expression are similar in the other E-cadherin-positive cell lines (HTB-126, HTB-27) examined (Figure 2D,E). However, in the E-cadherin-negative cell lines (MDA-MB-468, MDA-MB-231, BT-549), while the time course of increased expression over the first 4 h followed by a decrease is similar, the peak levels of expression are far less (*p* < 0.01) (Figure 2F–H). These results suggest that E-cadherin expression may play an important role in the full upregulation of HIF-1α during the early stages of 3D spheroid formation.

### 3.3. Increased HIF-1α Expression in Induced 3D Spheroidgenesis Is Not Mediated by Altered HIF-1α Hydroxylation nor by Altered VHL-Mediated Degradation

**Figure 3:**
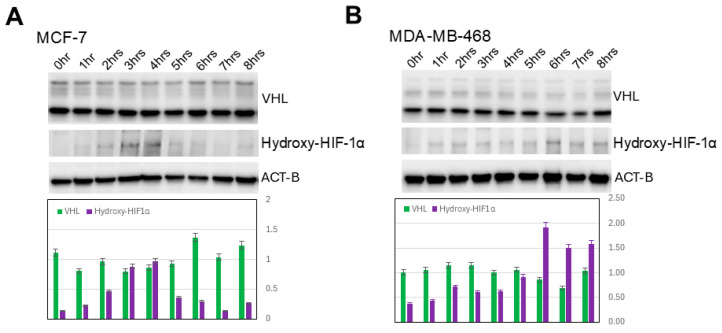
Lack of hydroxylation and VHL-mediated degradation of HIF-1α. Western blot analysis shows lack of increase in either HIF-1α hydroxylation/molecule or VHL along indicated time course in either E-cadherin-positive (**A**) or -negative cell lines (**B**).

While the levels of HIF-1α hydroxylation parallel the increase and decrease in overall levels of HIF-1α previously demonstrated over the 0–8 h time course, there is no significant increase in the degree of HIF-1α hydroxylation per HIF-1α molecule (*p* > 0.1) nor is there any significant increase or decrease in VHL levels over this same time period (*p* > 0.1) (Figure 3). This is true in all the E-cadherin-positive as well as -negative cell lines studied (Figure 3A,B).

### 3.4. Increased HIF-1α Expression in Induced 3D Spheroidgenesis Is Mediated in Small Part by Notch1 Signaling

**Figure 4:**
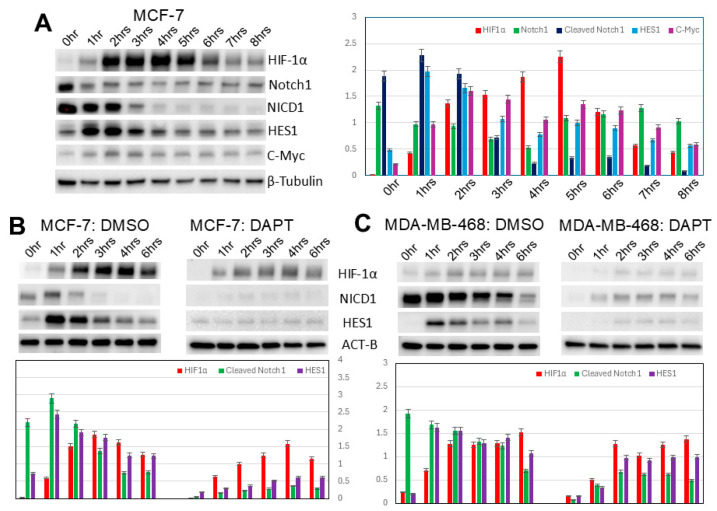
Notch1 signaling in induced spheroidgenesis. Western blot analysis shows bimodal Notch1 activation pattern both immediately and after 4 h with downstream signaling (**A**–**C**), both inhibited by DAPT. This Notch1 signaling increases HIF-1α levels in both E-cadherin-positive as well as -negative cell lines (**B**,**C**), but the Notch 1 signaling is greater in E-cadherin-negative cell lines (**B**,**C**).

Both increased Notch1 and increased Notch1 signaling occur both immediately (*p* < 0.01) and belatedly (after 4 h) (*p* < 0.05) (Figure 4A). Two of the canonical targets of Notch1, HES1 and c-Myc, are also expressed in a similar time sequence (*p* < 0.05) (Figure 4A). Predictably, Notch1 inhibition with DAPT abolishes both the immediate and belated Notch1 signaling (Figure 4B). Notch1 signaling is much lower and decreases more with Notch1 inhibition in E-cadherin-positive (*p* < 0.01) vs. E-cadherin-negative cell lines (*p* < 0.05) (Figure 4B,C). These findings suggest that E-cadherin partially inhibits Notch1 signaling. Notch1 signaling increases HIF-1α levels (*p* < 0.01), which are lowered by Notch1 inhibition with DAPT (Figure 4B,C). While the overall increase in HIF-1α is significantly greater in E-cadherin-positive cell lines (*p* < 0.01), the relative effects of Notch1 signaling are greater in E-cadherin-negative cell lines (*p* < 0.05) (Figure 4B,C). This suggests that E-cadherin primarily regulates HIF-1α not through Notch1 signaling but through other, more dominant signaling pathways.

### 3.5. Increased HIF-1α Expression in Induced 3D Spheroidgenesis Is Mediated in Large Part by mTOR Signaling

**Figure 5:**
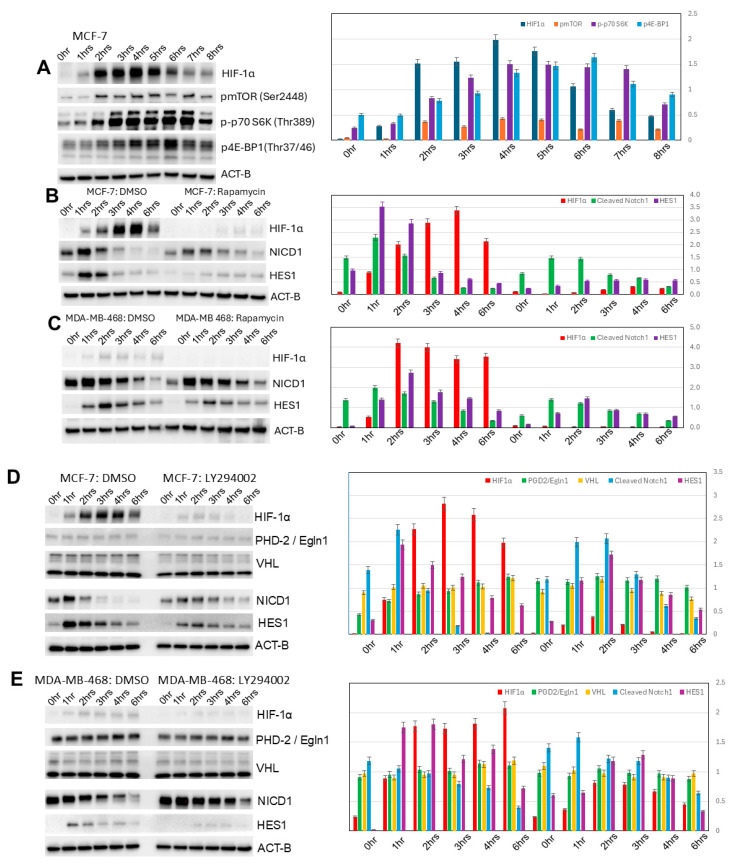
PI3K/AKT/mTOR signaling in induced spheroidgenesis. Western blot analysis shows mTOR activation and signaling during induced spheroidgenesis, which parallels increases in HIF-1α levels (**A**). Both mTOR signaling and HIF-1α levels are greater in E-cadherin-positive cell lines than -negative cell lines and are significantly inhibited by rapamycin, but Notch1 signaling is lower (**B**,**C**). Western blot also shows that PI3K/AKT signaling increases HIF-1α levels more in E-cadherin-positive cell lines than -negative cell lines (**D**,**E**). This inhibitor does not appreciably affect Notch1 signaling nor alterations in either PHD-2/Egln1 or VHL (**D**,**E**).

Levels of p-mTOR (Ser2448), p-p70S6K (Thr389), and p-4E-BP1 (Thr37/46) all increase in induced MCF-7 3D spheroidgenesis (*p* < 0.01), correlating with the upregulated expression of HIF-1α (*p* < 0.01) (Figure 5A). Rapamycin, an mTOR inhibitor, blocks the expression of HIF-1α in early induced MCF-7 spheroidgenesis (*p* < 0.01) (Figure 5B). A similar pattern of increased mTOR signaling correlating with increased HIF-1α and its inhibition with rapamycin is observed during induced 3D spheroidgenesis in E-cadherin-negative cell lines (*p* < 0.05) (Figure 5C). The only differences between the E-cadherin-positive and -negative cell lines were the overall higher levels of HIF-1α (*p* < 0.01) and the greater inhibition by rapamycin in the E-cadherin-positive lines (*p* < 0.05) (Figure 5B,C). This suggested that E-cadherin increases HIF-1α through increased mTOR signaling.

### 3.6. Increased HIF-1α Expression in Induced 3D Spheroidgenesis Is Mediated in Large Part Also by Phosphoinositide 3-Kinase (PI3K) Signaling

Inhibition of PI3K signaling with LY294002 results in a greater decrease in HIF-1α levels in induced 3D spheroidgenesis of E-cadherin-positive (*p* < 0.01) (Figure 5D) vs. E-cadherin-negative cell lines (*p* < 0.05) (Figure 5E). This P13K inhibition does not alter the levels of PHD-2/Egln1 or VHL (*p* > 0.1) (Figure 5D,E). LY294002 does not appreciably affect Notch1 signaling (*p* > 0.1). These findings suggest that E-cadherin increases HIF-1α levels through increased PI3K signaling.

### 3.7. Decreased HIF-1α Expression in Induced 3D Spheroidgenesis Is Observed with E-Cadherin Knockout

**Figure 6:**
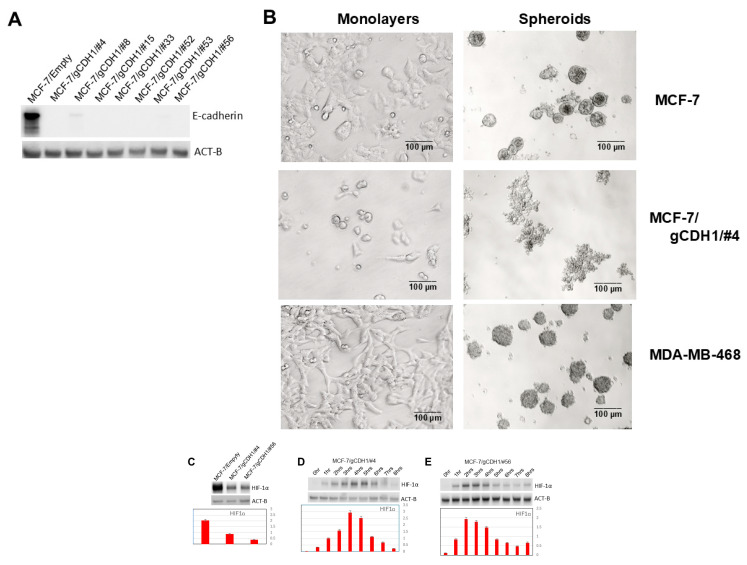
Effects of E-cadherin gene knockout on induced spheroidgenesis, HIF-1α, and downstream signaling. Western blot shows the absence of E-cadherin in select knockout MCF-7 clones (**A**) and the phenotypic effects of this knockout on the appearance of both the monolayers as well as the induced spheroids with both the wild type E-cadherin-positive MCF-7 as well as the E-cadherin-negative MDA-MB-468 (illustrated for comparison). Scale bars are provided (**B**). MCF-7 E-cadherin knockdown significantly decreases overall HIF-1α levels (**C**) but does not alter the expression pattern over the time course of induced spheroidgenesis (**D**,**E**).

As mentioned, our results with the mTOR and P13K signaling experiments in E-cadherin-positive vs. -negative cell lines suggest that E-cadherin is promoting high levels of HIF-1α expression in induced spheroidgenesis. To prove and further corroborate the role of E-cadherin in increasing HIF-1α in induced spheroidgenesis, we employ the CRISPR-Cas9 system to generate E-cadherin-knockout MCF-7 clones (Figure 6A). These E-cadherin-knockout clones, compared phenotypically to both wild-type MCF-7 cells and E-cadherin-negative cell lines (e.g., MDA-MB-468), show decreased adhesion and spreading in monolayer culture and a dramatic decrease in both density and cell-to-cell tightness in suspension culture (Figure 6B). Although HIF-1α levels are markedly reduced in the E-cadherin-knockout clones (*p* < 0.01) (Figure 6C), the time course of HIF-1α expression (Figure 6D,E) was no different than that exhibited by wild-type MCF-7 (*p* > 0.1). This suggests that while E-cadherin overall increases HIF-1α levels, the pattern of HIF-1α expression over time is regulated by the stage of induced spheroidgenesis and not by E-cadherin.

### 3.8. Decreased HIF-1α Expression in Induced 3D Spheroidgenesis Is Observed Due to Calpain Activity

**Figure 7:**
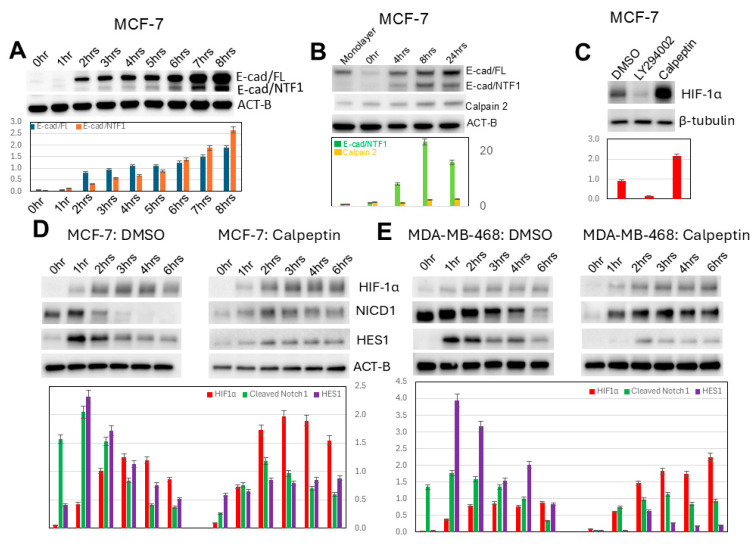
Effects of calpain on induced spheroidgenesis, HIF-1α, and downstream signaling. E-cadherin proteolysis, generating E-cad/NTF1, occurs during induced spheroidgenesis (**A**). During induced spheroidgenesis, both calpain 2 and calpain-mediated E-cadherin proteolysis (E-cad/NTF1) increase, peaking around 8 h and then mildly decrease (**B**). Inhibition by calpeptin significantly increases HIF-1α expression, while inhibition by LY294002 decreases HIF-1α expression (**C**). Calpeptin dramatically increases HIF-1α levels (**C**) in both E-cadherin-positive (**D**) as well as -negative cell lines (**E**). Calpeptin significantly decreases Notch1 signaling in both E-cadherin-positive (**D**) as well as -negative cell lines (**E**), with the decrease in Notch signaling being greater in E-cadherin-negative cell lines (**D**,**E**).

Both E-cadherin and its calpain-generated intracellular fragment, E-cad/NTF1, increase during induced MCF-7 spheroidgenesis (Figure 7A), peaking around 8 h (*p* < 0.01) and only mildly decreasing at 24 h (Figure 7B). During this induced spheroidgenesis, increased E-cadherin proteolysis (E-cad/NTF1) correlates with increased calpain 2 (*p* < 0.01) (Figure 7B). The levels of both E-cadherin and E-cadherin proteolysis certainly cannot explain the decrease in the expression of HIF-1α at these later stages (8 h) of induced spheroidgenesis. Inhibition of calpain activity by calpeptin significantly increases HIF-1α expression (*p* < 0.01) while inhibition of P13K with LY294002 decreases HIF-1α expression (*p* < 0.01) (Figure 7C). For these latter experiments, we used a time point of 4 h after seeding because this is the time point at which induced spheroidgenesis shows peak levels of HIF-1α. Therefore, the inhibitory effects of LY294002 and the stimulatory effects of calpeptin could be observed at the time point where untreated induced spheroidgenesis shows peak levels of HIF-1α.

Our results also show that calpeptin increases HIF-1α expression in both E-cadherin-positive (*p* < 0.05) (Figure 7D) and E-cadherin-negative cell lines (*p* < 0.01) (Figure 7E). Since the latter lack E-cadherin and, therefore, calpain-mediated E-cadherin proteolysis, the effects of calpeptin on increased HIF-1α expression must occur through both calpain-mediated proteolysis of E-cadherin as well as calpain-mediated proteolysis of other substrates. Unlike the inhibition of PI3K/AKT signaling with LY294002, which did not affect Notch1 signaling (*p* > 0.1), the inhibition of calpain with calpeptin significantly decreases Notch1 signaling in all lines (*p* < 0.05) (Figure 6D,E) but more so in E-cadherin-negative ones (*p* < 0.01) (Figure 6E).

## 4. Discussion

The catalyst behind the development of 3D models as a replacement for traditional 2D models was the overall failure of 2D cell culture models to provide translational therapeutic insights into cancer biology and behavior occurring in patients [1]. Because of a desire to reproduce in vitro, or at least in experimental animals, the autocrine and paracrine cellular interactions occurring in patients (which would better guide therapy), different types of 3D models have arisen over the past several decades [1]. These have included both scaffold-containing and scaffold-free models, the latter including tumoroids, organoids, and spheroids [3,31,32]. Spheroids consist of clusters of tumor cells that come into immediate proximity to one another in a 3D configuration [33]. Oftentimes, this 3D configuration stimulates the cells to form tight junctions, gap junctions, and desmosomes, mirroring the structures observed within cancers in patients [34]. Drug sensitivity studies further highlight the advantages of 3D systems over traditional 2D cultures. Tumor cells in spheroids are less sensitive to chemotherapeutic agents due to barriers in drug penetration, hypoxia-driven cellular changes, and altered cell cycle dynamics that more accurately reflect in vivo tumors [35].

Spontaneous vs. induced 3D spheroidgenesis differ regarding their E-cadherin dependency. Although there are very few breast cancer models of spontaneous spheroidgenesis, certain spontaneous spheroidgenesis models, like Mary-X, self-assemble into compact spheroids without external scaffolds, reflecting intrinsic cellular programs driven by adhesion molecule expression such as E-cadherin, cytoskeletal dynamics, and genetic programs associated with metastasis [36]. In contrast, all breast cancer cell lines studied to date can be induced to undergo 3D spheroidgenesis and, during induced spheroidgenesis, activate the signaling pathways described in the present study. While E-cadherin regulates the signaling that characterizes 3D spheroidgenesis, the presence or absence of E-cadherin can be independent of ER, PR, or Her-2/neu status, and hence the observations made in the present study can be applied to all of the traditional molecular subtypes of human breast cancer. Although numerous studies have described the spatiotemporal relevance of these 3D models [37,38,39], there has been a paucity of studies investigating the actual signaling pathways that are activated during spheroidgenesis. Since both in vitro 3D spheroidgenesis and its in vivo equivalents undoubtedly involve common cell adhesion, growth, metabolism, and hypoxia-regulating pathways [26,27,28,29,30], which were also observed in our previous studies of spontaneous spheroidgenesis [23,24,25], we focused on these in the present study.

Increased HIF-1α expression in induced 3D spheroidgenesis is upregulated by E-cadherin. Emerging studies highlight that HIF-1α activation in induced 3D spheroid systems is not solely oxygen-dependent. Although hypoxia plays a critical role in cancer biology, common hypoxia-related signaling pathways can also be triggered under conditions of normoxia. In the present study, increased HIF-1α expression noted in early 3D spheroidgenesis occurs under normoxia. The initial phase of spheroidgenesis can be characterized by cellular interactions, which may involve signaling pathways that lead to changes in HIF-1α levels, even before a hypoxic core develops within the spheroid. For example, in other recent studies, reactive oxygen species (ROS) have been implicated in early aggregation events during induced MCF-7 spheroid formation, and ROS, in turn, can influence HIF-1α transcription, leading to transient HIF-1α upregulation in the initial stages of spheroid formation [40,41].

Increased HIF-1α expression in induced 3D spheroidgenesis is not mediated by altered HIF-1α hydroxylation nor by altered VHL-mediated degradation. Under normoxic conditions, HIF-1α is tightly controlled by VHL protein, a tumor suppressor that acts as part of an E3 ubiquitin ligase complex. Hydroxylation of HIF-1α by prolyl hydroxylase domain enzymes allows for recognition by VHL, which mediates polyubiquitination and subsequent proteasomal degradation. In spheroid cultures experiencing hypoxia, hypoxia rapidly stabilizes HIF-1α due to impaired hydroxylation, leading to its nuclear accumulation [42,43,44,45,46,47]. But in early spheroidgenesis under normoxia, both the stability and activity of HIF-1α can be regulated through various mechanisms, all independent of altered HIF-1α hydroxylation or VHL-mediated degradation [48]. For instance, growth factors, cytokines, and oncogenic signaling pathways can induce HIF-1α expression and activity regardless of oxygen concentration [49].

Increased HIF-1α expression in induced 3D spheroidgenesis is mediated in small part by Notch1 signaling. Since it had been previously observed that there is a complex crosstalk between both HIF-1α and the Notch signaling pathway, and that activation of Notch signaling upregulates HIF-1α expression through HES1 (a key transcriptional repressor and downstream target of Notch signaling [27,50,51,52,53,54]), we investigated this in our models of induced 3D spheroidgenesis. Both increased Notch1 and increased Notch1 signaling occur both immediately and belatedly in induced spheroidgenesis. This immediate increase in Notch1 and Notch1 signaling is completely independent of induced spheroidgenesis as it occurs before induced spheroidgenesis is initiated and has been attributed to trypsin’s detergent actions, which can stress cells as they are disrupted from monolayer cultures and can even upregulate HIF-1α [55]. However, it is the belated Notch1 signaling (after 4 h) that is related to induced 3D spheroidgenesis. A crosstalk between Notch1 and HIF-1α, potentially through the NF-κB pathway, has been shown to enhance stemness properties, promote anoikis resistance, and facilitate the self-assembly of spheroids under low-attachment conditions [56].

Increased HIF-1α expression in induced 3D spheroidgenesis is mediated in large part by mTOR signaling. Since the mammalian target of rapamycin (mTOR) plays a significant role in the regulation of HIF-1α expression [57,58], we decided to investigate mTOR signaling in our models of induced 3D spheroidgenesis. In our previous studies, we directly demonstrated that rapamycin inhibits mTOR and mTOR signaling in spontaneous spheroidgenesis [24,25]. And in the present study, rapamycin decreased HIF-1α in both E-cadherin-positive and -negative cell lines. Therefore, it was reasonable to conclude that the HIF-1α inhibition was mediated through mTOR inhibition. Several studies have demonstrated that mTOR signaling promotes the synthesis of HIF-1α protein, independently of oxygen status. In spheroid cultures, where nutrient gradients and metabolic reprogramming are pronounced, mTOR activity supports the persistence of HIF-1α, thereby maintaining hypoxia-responsive gene expression. Inhibition of mTOR has been shown to reduce spheroid viability [59].

It is important to emphasize that our present study focuses on the early stages of induced spheroidgenesis. One of the main findings is that HIF-1a is upregulated in the early stages of spheroidgenesis: its level increases and peaks at 4 h; after that time, its level decreases. In a previous study [24], we had shown that in the early stage of spheroidgenesis (0 to 8 h) of both E-cadherin-positive and -negative cell lines, mTOR is activated in a similar pattern: mTOR activity increases and reaches its highest level at 4 h and then decreases and reaches its lowest level at 8 h. Therefore, this period really is the perfect window to compare the differences in the regulation of HIF-1a in induced spheroidgenesis. In our present study, the inhibition of mTOR led to the downregulation of HIF-1a in both E-cadherin-positive cells and E-cadherin-negative cells. The difference is that HIF-1a is upregulated more so in E-cadherin-positive cells than in E-cadherin-negative cells.

Increased HIF-1α expression in induced 3D spheroidgenesis is mediated in large part also by phosphoinositide 3-kinase (PI3K) signaling. Since the PI3K/AKT/mTOR pathway is a known signaling cascade that plays a significant role in regulating cellular processes, including HIF-1α expression [60,61,62,63,64,65,66,67], and since in previous studies, we reported on the role of P13K in regulating metabolism through mTOR signaling in spontaneous spheroidgenesis [24,25], we decided to investigate P13K and HIF-1α expression in induced spheroidgenesis. The phosphoinositide 3-kinase (PI3K) pathway represents another upstream regulator of HIF-1α stabilization and transcriptional activity. PI3K signaling enhances HIF-1α via activation of AKT and downstream mTOR, leading to increased translation and reduced degradation. Pharmacological inhibition of PI3K has been reported to disrupt spheroid formation and re-sensitize cells to therapy, underscoring its importance in maintaining HIF-1α-dependent survival mechanisms [68,69].

Decreased HIF-1α expression in induced 3D spheroidgenesis is observed in E-cadherin knockout. Observing this phenomenon under normoxic conditions is highly novel. It was previously believed that the functional relationship between HIF-1α and E-cadherin under hypoxic conditions was reciprocal and central to spheroidgenesis. On one hand, HIF-1α promotes EMT by upregulating transcription factors, such as SNAIL, TWIST, and ZEB1, which suppress E-cadherin transcription, weaken adherent junctions, and increase migratory potential [70,71,72]. On the other hand, the loss of E-cadherin itself may indirectly stabilize HIF-1α, as reduced cell–cell adhesion enhances hypoxia responses and alters intracellular signaling [73]. This mutual regulation highlights E-cadherin as a downstream target of HIF-1α. Our studies indicate the opposite: HIF-1α is a downstream target of E-cadherin and is downregulated with E-cadherin knockout.

Decreased HIF-1α expression in induced 3D spheroidgenesis is observed due to increased calpain 2 activity. Calpains, a family of calcium-dependent cysteine proteases, have recently emerged as regulators of HIF-1α stability and activity. Calpain, particularly the calpain-2 isoform, plays a critical role in promoting HIF-1α nuclear translocation and regulating its activity by interfering with its degradation pathways, cleavage of filamin A, and by activating PI3K/AKT signaling, which in turn influences metastasis [74,75]. In spheroid models, elevated calpain activity has been associated with both increased HIF-1α levels and the acquisition of EMT traits, including suppression of E-cadherin. Targeting calpain activity has been proposed as a potential strategy to reduce HIF-1α–mediated tumor aggressiveness [76]. In our induced models of spheroidgenesis, our observations are the opposite and novel: inhibition of calpain by calpeptin increases HIF-1α. This inhibition by calpeptin would increase the steady state levels of full-length E-cadherin, which would be the opposite of the effects of E-cadherin knockout, which results in decreases in HIF-1α levels. Predictably, then, calpeptin should increase HIF-1α levels, which was observed.

Our results also show that calpeptin increases HIF-1α expression in both E-cadherin-positive and E-cadherin-negative cell lines. Since the latter lack E-cadherin and therefore calpain-mediated E-cadherin proteolysis, the effects of calpeptin on increased HIF-1α expression must occur through both calpain-mediated proteolysis of E-cadherin as well as calpain-mediated proteolysis of other substrates. Unlike inhibition of PI3K/AKT signaling with LY294002, which did not affect Notch1 signaling, inhibition of calpain with calpeptin significantly decreases Notch1 signaling in all lines, but more so in E-cadherin-negative ones. This suggests that calpeptin might cross-inhibit other proteases, like γ-secretase, which cleaves Notch1 and activates Notch1 signaling. Obviously, a greater understanding of the signaling pathways that are activated as a result or product of induced 3D spheroidgenesis is needed.

## Data Availability

Both Mary-X and the other cell lines used in this study are available to any investigator upon request. These include all of the E-cadherin MCF-7 knockout clones. All data sets generated and used in the study are also available upon request.
